# An Unusual Sticky Situation of New-Onset Right Sided Heart Failure

**DOI:** 10.15761/jccr.1000168

**Published:** 2021-05-17

**Authors:** Demetrio Sharp Dimitri, Priya V Parikh, Sneha Sharma, Sven Wang, Ahmed Souka, Mohamed Effat

**Affiliations:** 1 Department of Internal Medicine, University of Cincinnati Medical Center, USA; 2 University of Cincinnati College of Medicine, USA; 3 Department of Internal Medicine, Division of Cardiology, Interventional Cardiology, University of Cincinnati Medical Center, USA

**Keywords:** hemodynamics, constrictive pericarditis, pericardial effusion, right-sided catheterization autoimmune, imaging, pulmonary hypertension, pressure waves, cardiac physiology

## Abstract

This case highlights the importance of having constrictive pericarditis (CP) as a differential diagnosis in unexplained sign and symptoms of right-sided heart failure. This case portrays challenges in diagnosing CP caused by certain rheumatologic diseases despite advances in diagnostic modalities, clinical suspicion remains the most important tool for this diagnosis.

## Introduction

Constrictive pericarditis (CP) is a rare condition that often presents with non-specific symptoms and is often misdiagnosed. CP is characterized by a rigid pericardium that restricts the heart, resulting in signs and symptoms of diastolic heart failure. It occurs as a late sequela of pericardial inflammation caused by prior infection or radiation, inflammatory diseases and rarely, connective tissue disorders. We present a case of new-onset right-sided heart failure caused by constrictive pericarditis in a patient with diffuse systemic sclerosis (SSc). This case highlights the importance of having CP in our differential diagnosis in patients with unexplained sign and symptoms of right-sided heart failure and increased systemic venous pressure. This is crucial since CP secondary to SSc is largely curable and failure to diagnosis has devastating consequences. This case portrays the challenges in diagnosing pericardial diseases caused by certain rheumatologic diseases and that despite advances in diagnostic modalities, clinical suspicion remains the most important tool for this diagnosis.

## Case

A 27-year-old female with a seven-year history of diffuse systemic scleroderma complicated by recurrent pericarditis and severe Raynaud’s disease resulting in gangrene and amputation of the upper and lower extremity digits presented to the hospital with two weeks of progressive shortness of breath, early satiety, fatigue, abdominal distention, and lower extremity pain and edema. The physical examination was notable for the presence of jugular vein distention with positive hepatojugular reflux, right upper quadrant tenderness, ascites and 3+ lower extremity edema to the thighs. Her heart rate was 108 beats per minute; other vital signs were within normal limits. EKG showed sinus tachycardia with nonspecific ST-T wave changes. Chest x-ray showed a moderate right-sided pleural effusion with right basilar airspace opacity and a cardio mediastinal silhouette. Transthoracic echocardiogram demonstrated a normal left ventricular ejection fraction, mildly reduced right ventricular function, mild pulmonary hypertension with an estimated mean pulmonary artery pressure of 40 mmHg, and no significant valvular abnormalities. Abdominal ultrasound redemonstrated ascites and was also notable for hepatomegaly and a hyperechoic pancreatic mass.

**Figure 1.**
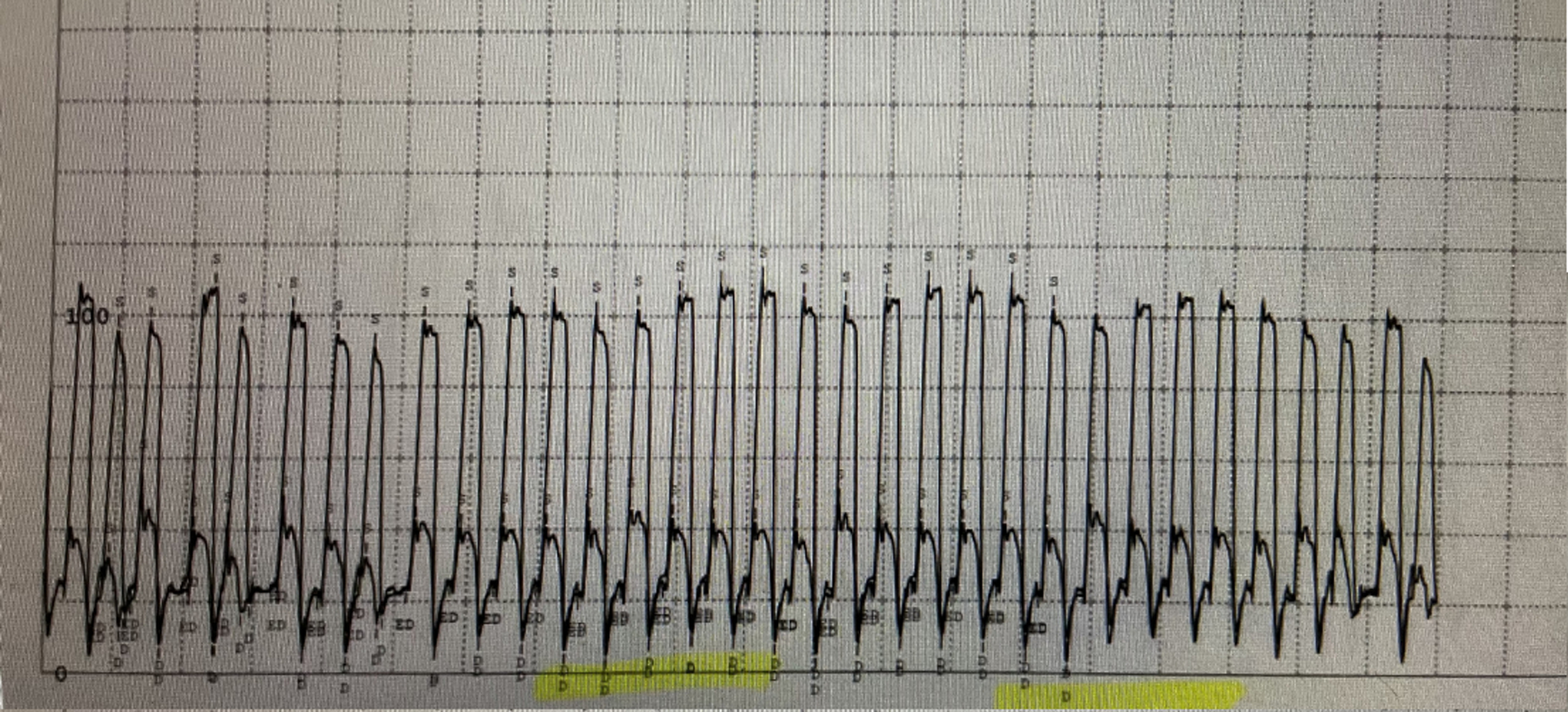
Intracardiac Pressure Tracing of Constrictive Pericarditis. Ventricular Interdependence

**Figure 2.**
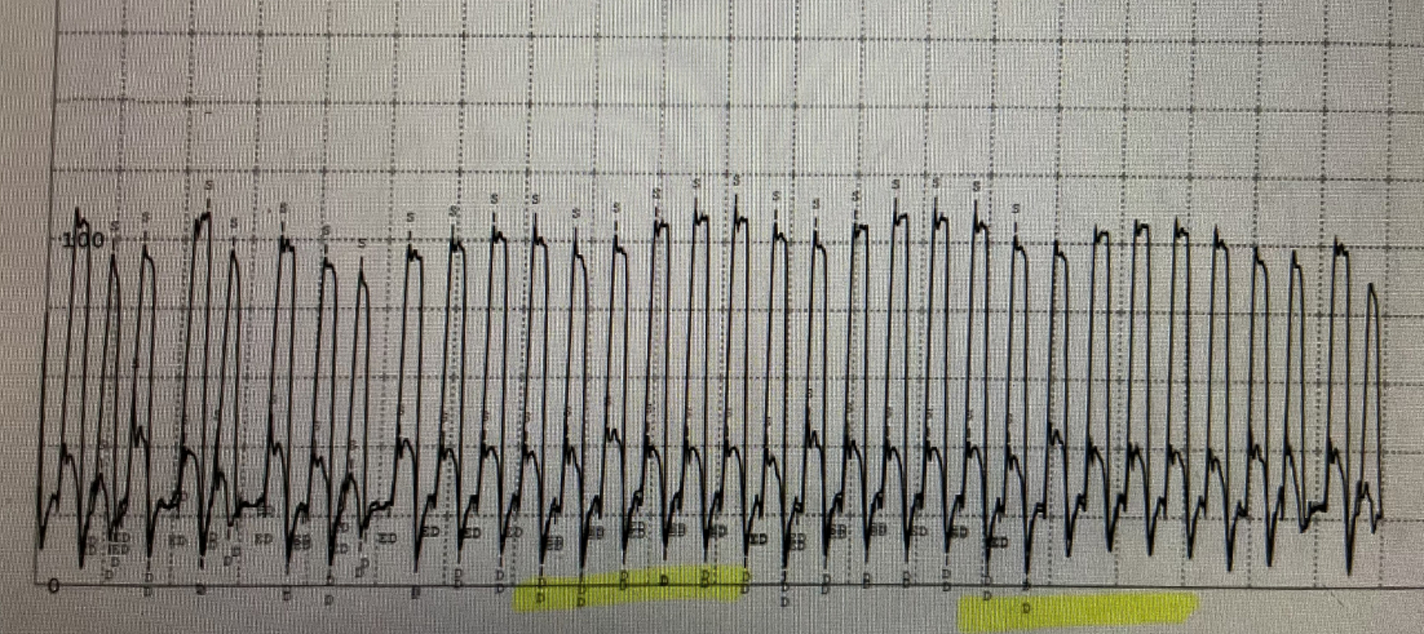
Right ventricular and left ventricular pressure trace shows ventricular discordance. During inspiration peak systolic pressure in LV is reduced with corresponding increase in RV pressure they move in opposite direction. Exactly reverse happens during expiration

**Figure 3.**
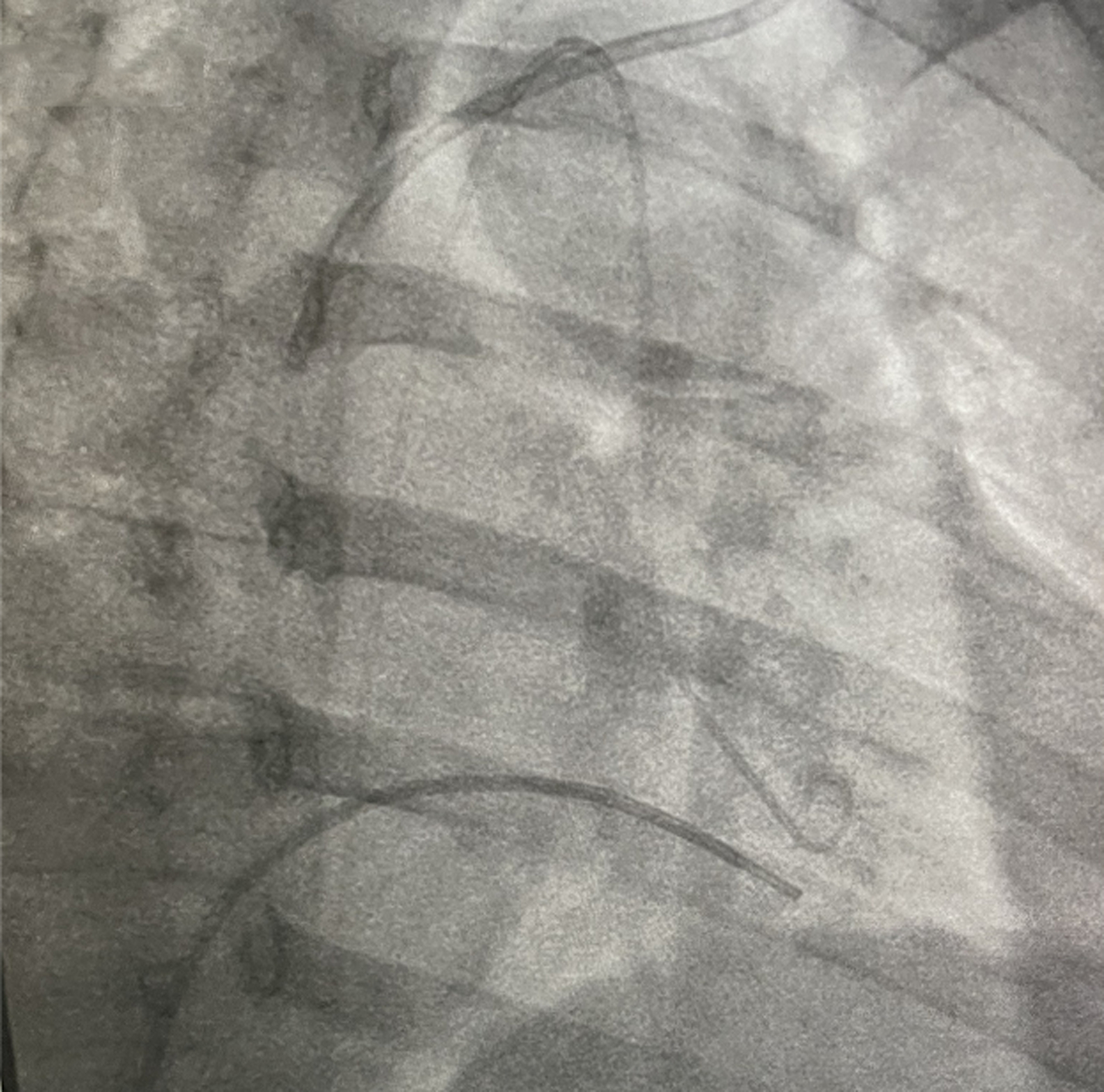
Simultaneously recording pressure of RV and LV to prove ventricular interdependence

Initially the patient’s ascites was attributed to intrinsic hepatic disease and she was started on diuretics. However, her renal function deteriorated, halting diuresis while further diagnostic evaluation was pursued. The patient continued to have abdominal distension and shortness of breath which prompted a cardiovascular evaluation with a right heart catheterization. Mild pulmonary arterial hypertension was noted with elevated mean pulmonary arterial pressure (mPAP) of 28 mmHg. Due to the discrepancy between the mildly elevated cardiac filling pressures and significant anasarca on physical examination, a repeat hemodynamic assessment was pursued. The patient underwent right and left heart catheterization which discovered near equalization of the diastolic filling pressures, raising suspicion for pericardial tamponade constrictive physiology (Table 1). In addition, the data demonstrated an abrupt decrease in early diastolic pressures followed by a rapid increase then plateau of the left and right ventricular pressures (square root sign) with subsequent ventricular interdependence (Figures 1–3).

As most diagnostic imaging studies were contraindicated due to the patient’s impaired renal function, a cardiac MRI without contrast was obtained to evaluate for possible pericardial disease. Cardiac MRI showed an abnormally thick pericardium (4–5 mm) and evidence of significant pericardial adhesion with a small dependent pericardial effusion. The ventricles were cone shaped and a septal bounce was seen during diastole. In addition, free breathing CINE imaging demonstrated ventricular interdependence. These findings were consistent with pericardial constriction.

## Management

After extensive discussion between cardiology, rheumatology, nephrology and cardiothoracic surgery, the primary team agreed that surgical intervention was the best option to improve this patient’s quality of life and disease prognosis. Subsequently, she underwent successful pericardiectomy with complete resolution of her symptoms. The pathology report revealed extensive dense fibrosis in the pericardium that was likely secondary to patient’s known collagen vascular disease.

## Discussion and conclusion

Cardiac involvement in both limited and diffuse SSc has been recognized over the years as one of the most rare and feared complications due to poor prognosis [1]. Cardiac involvement is clinically apparent in 10–30% of patients with systemic sclerosis and can manifest in a variety of ways, including pericarditis, pericardial effusion, myocardial fibrosis, myocardial infarction, diastolic heart failure, conduction system abnormalities, arrhythmias and sudden death. Once the disease involves the heart, the mortality rate increases to approximately 60–75% at five years [2]. Pericardial involvement can be especially deadly in these patients as it is seen in up to 77% of postmortem biopsies [3]. Subclinical cardiac manifestations are estimated at over 70% and rely significantly on a strong clinical suspicion and diagnostic tools [4].

The noteworthy pathophysiology involved in presentation of this condition is unique in that there is recurrent coronary microvascular ischemia and myocardial inflammation which leads to ischemic necrosis, reperfusion damage, and myocardial fibrosis [5]. This adverse remodeling results in a stiffened pericardium which causes dissociation of intrathoracic and intracardiac pressures. This dissociation results in loss of interventricular interdependence and impaired left ventricular output during inspiration, manifested as characteristic findings on physical examination (Kussmaul sign) and echocardiography (septal bounce).

However, constrictive physiology can complicate virtually any condition associated with pericardial effusion [6]. The non-specificity of the presenting symptoms such as abdominal discomfort and ascites have historically led to late recognition or even misdiagnosis of this condition, resulting in unnecessary procedures. Consequently, it is imperative to consider this diagnosis when evaluating a patient with sign and symptoms of diastolic heart failure and venous congestion as this is one the few “curable” causes of these disorders.

Certain serologic tests for patients with systemic sclerosis can provide useful information about specific organ involvement, including cardiac manifestations. Studies have shown that patients with SSc with myocarditis had cytoplasmic antineutrophil cytoplasmic antibody (c-ANCA) and antiproteinase 3 (PR3) antibody positivity [7]. Furthermore, an association between cardiac involvement and the presence of anti-topoisomerase and anti-U3-RNP antibodies has been indicated [8]. Despite evidence showing serologic association with cardiac manifestations in patients with SSc, initial baseline and annual cardiac screening should be done in all of these patients, including a focused history and physical exam, an electrocardiogram (ECG), measurement of natriuretic peptides, and cardiac troponin. Additional cardiac testing, such as a cardiac MRI or cardiac catheterization can be done if initial testing is suspicious of cardiac involvement.

Overall, due to the high prevalence of cardiac involvement in patients with SSc, it becomes imperative to do a thorough history taking and examination of this population. Prompt recognition and management of constrictive pericarditis can alleviate symptoms such as volume overload, drastically transform these patients’ quality of life, improve prognosis, and decrease mortality.

## Learning objectives

A patient presenting with an odd case of Constrictive Pericarditis due to Systemic Sclerosis.

1. This case portrays the challenges in diagnosing pericardial diseases caused by certain rheumatologic diseases and that despite advances in diagnostic modalities, clinical suspicion remains the most important tool for this diagnosis.
2. To be able to recognize rare but very specific findings of a constrictive pericarditis physiology.
3. To understand the role of Multidisciplinary approach to benefit the patient’s quality of life.

## Figures and Tables

**Table 1.** Intracardiac Pressures Evidencing Equalization of All Cardiac Chambers During Diastole

	RHC #1	RHC#2 + LHC
Right Atrial Pressure	16	16
Right Ventricle Pressure	39/16	45/22
Pulmonary Artery	39/17	45/20
Pulmonary Capillary Wedge (PCWP)	17	20
Cardiac Output (Fick)	6.9 L/min	6.8 L/min
Cardiac Index (Fick)	3.8 L/min/1.23m2	3.7l/min/
Systemic Vascular Resistance (SVR)	887	993
Pulmonary Vascular Resistance (PVR)	104	118
Left Ventricular End Diastolic Pressure (LVEDP)		20
